# The Impact of Lighting Treatments on the Biosynthesis of Phenolic Acids in Black Wheat Seedlings

**DOI:** 10.3390/foods13162499

**Published:** 2024-08-09

**Authors:** Hongjie Lan, Chunping Wang, Zhengfei Yang, Jiangyu Zhu, Weiming Fang, Yongqi Yin

**Affiliations:** College of Food Science and Engineering, Yangzhou University, Yangzhou 210095, China; mz120232122@yzu.edu.cn (H.L.); mz120211961@yzu.edu.cn (C.W.); yzf@yzu.edu.cn (Z.Y.); 008051@yzu.edu.cn (J.Z.); wmfang@yzu.edu.cn (W.F.)

**Keywords:** phenolic acid, black wheat, red light, ultraviolet, germination

## Abstract

Light, as a crucial environmental determinant, profoundly influences the synthesis of secondary metabolites in plant metabolism. This study investigated the impacts of the red light combined with ultraviolet-A (UV-A) and ultraviolet-B (UV-B) treatments on phenolic acid biosynthesis in black wheat seedlings. The results demonstrate that the red light combined with UV-A and UV-B treatments significantly enhanced the levels of phenolic acids in black wheat seedlings, at 220.4 μg/seedling and 241.5 μg/seedling, respectively. The content of bound phenolic acids in black wheat seedlings increased by 36.0% under the UV-B treatment. The application of the UV-A/UV-B treatments markedly enhanced the activities of phenylalanine ammonia-lyase, 4-coumarate CoA ligase, and cinnamate 4-hydroxylase in black wheat seedlings while also promoting the expression levels of genes related to phenolic acid synthesis. The expression levels of *fructose-1,6-bisphosphate aldolase* and *NADP-malic enzyme* related to photosynthesis were significantly upregulated. This resulted in an augmentation in the chlorophyll content, thereby enhancing photosynthesis in black wheat seedlings. Nevertheless, the UV-A and UV-B treatments also had a significant constraining effect on the growth and development of black wheat seedlings. In addition, the UV-A and UV-B treatments increased the activity and gene expression levels of antioxidant enzymes while significantly increasing the contents of total flavonoids and anthocyanins, activating the antioxidant system. The findings reveal that light-source radiation serves as an effective method for promoting the biosynthesis of phenolic acids in black wheat seedlings.

## 1. Introduction

Wheat (*Triticum aestivum* L.), an ancient food crop, is a significant source of energy for humans, accounting for 20% of the global dietary intake [1]. Wheat is not only rich in protein, vitamins, and a variety of mineral elements but, more importantly, its phenolic acid content is higher than that of other grain crops [2]. Phenolic acids represent a category of plant secondary metabolites distinctively marked by the attachment of hydroxyl to aromatic rings. This class encompasses compounds such as caffeic acid, vanillic acid, and ferulic acid. They exhibit numerous diverse physiological effects on the human body, such as anti-oxidation, anticancer, anti-inflammatory, and antihypertension [3]. Extensive clinical research has validated that the inclusion of whole-grain wheat in one’s diet can notably decrease the risk of developing chronic diseases, including cancer and hypertension. This beneficial effect is primarily ascribed to the abundant presence of phenolic acid in wheat [4,5]. Amidst the escalating interest in the nutritional merits of food, there is great market potential in enhancing the contents of phenolic acids in wheat and developing foods rich in phenolic acids.

Phenolic acids in plants manifest in the following three distinct forms: free, bound, and conjugated. They mainly rely on the phenylpropanoid pathway to synthesize and metabolize [6]. Studies have demonstrated that wheat exhibits substantial elevations in the levels of ferulic acid and caffeic acid under drought treatment and heat treatment, respectively [7]. Similarly, low-temperature [8], salicylic acid [9], and methyl jasmonate [10] treatments also promoted the enrichment of phenolic acids in plants. This research substantiates that environmental conditions have an important impact on the biosynthesis of phenolic acids in plants. The phenylpropanoid pathway encompasses numerous pivotal enzymes that facilitate phenolic acid synthesis, with their activity and relative gene expression intricately tied to the content of phenolic acids [11]. NaCl treatment notably elevated the content of phenolic acids in barley by increasing the activities of phenylalanine ammonia-lyase (PAL), 4-coumarate-CoA ligase (4CL), and cinnamic acid 4-hydroxylase (C4H) and upregulating the expression levels of *PAL*, *4CL*, *C4H*, and *p-coumarate-3-hydroxylase* (*C3H*) [12]. Under a treatment of prohydrojasmon, the relative expressions of *PAL* and *flavanone 3-hydroxylase* (*F3H*) in lettuce increased, which promoted the enrichment of phenolic acids [13]. Exogenous salicylic acid treatment significantly enhanced the activities of PAL, 4CL, and C4H in kiwifruit and increased the relative expression of *PAL* and *4CL*, promoting the biosynthesis of phenolic acids [14]. The outcomes showed that environmental factors augment the enrichment of phenolic acids in plants by modulating the enzymatic activity and gene expression levels associated with phenolic acid biosynthesis.

As a crucial environmental factor in the life cycle of plants, light not only influences plant growth but also serves a critical function in modulating the production of secondary metabolites within plants. Research indicates that red light can promote the generation of phenolic acids in various vegetables, such as radish, wheat, and lentils [15]. Ultraviolet-A (UV-A) treatment notably increased the levels of phenols in strawberries [16] and broccoli [17]. Soybean sprouts exposed to ultraviolet-B (UV-B) irradiation significantly increased the isoflavone content [18]. In recent years, plants have been treated with light during germination, and changes in the content of secondary metabolites, especially phenols, have attracted people’s attention. Red light treatment significantly promoted the biosynthesis of ferulic acid and *p*-coumaric acid during wheat germination [19]. The phenolic acid content of kale was significantly increased by blue light treatment during germination [20]. The UV-B treatment markedly augmented the biosynthesis of phenolic acids in wheat [21] and barley [22] sprouts. This indicates that light treatment of germinated plants is a viable means to promote the biosynthesis of phenolic compounds in plants. Our prior investigations demonstrated that the red light combined with ultraviolet light treatment notably enhanced the progressive buildup of phenolic acids in wheat seedlings. Nevertheless, the underlying molecular mechanism responsible for the accumulation of phenolic acids in wheat seedlings, triggered by a treatment combining red light and ultraviolet light, remains poorly understood.

In this paper, the changes in physiology and active oxygen and phenolic acid contents during black wheat germination were analyzed by the red light combined with UV-A and UV-B treatments. The activities and gene expression changes of key enzymes in the phenolic acid synthesis pathway, antioxidant system, and photosynthetic system were investigated. This study elucidated the molecular mechanism for phenolic acid accumulation in black wheat seedlings induced by the combined treatment of red light and ultraviolet light. This study expands perspectives on the regulation of light in the phenolic metabolism of plants.

## 2. Materials and Methods

### 2.1. Germination Process and Experimental Design

**Figure 1:**
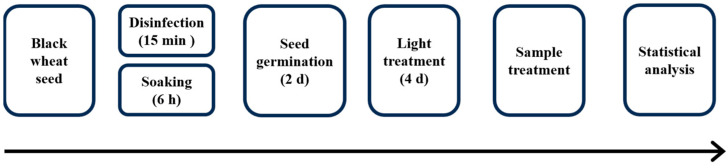
Schematic diagram of the treatments used to study the effects of the red light combined with UV-A and UV-B on black wheat seedlings.

The black wheat seeds (*Triticum aestivum* (L.) cultivar black vajra) were provided by Fuchun Seed Industry Co., Ltd., Qingdao, China. The seeds underwent sterilization in a 1% (*v*/*v*) sodium hypochlorite (NaClO) solution for 15 min, followed by rinsing with deionized water until neutrality was achieved. Subsequently, they were immersed in distilled water and maintained at 25 °C for a duration of 6 h. Following this, they were transferred onto germination plates and incubated at 25 °C in an incubator (PGX-150, Ningbo Haishu Saifu Experimental Instrument Co., Ltd., Ningbo, China). After 2 days of germination, according to our previous research, the following treatment groups were set: (1) CK—red light tubes were placed one foot above the germination tray (at 655 nm and with an intensity of 400 μmol·m^−2^·s^−1^, Shandong Guixiang photoelectric Co., Ltd., Shandong, China), cycled through 18 h of light and 6 h of darkness, and deionized water was sprayed. (2) UV-A—UV-A treatment (at 340 nm and with intensity of 40 μmol·m^−2^·s^−1^, Beijing Electronic Resource, Inc., Beijing, China) was applied on the basis of the red light treatment, cycled through 18 h of light and 6 h of darkness, and distilled water was sprayed. (3) UV-B—UV-B treatment (at a wavelength of 313 nm and intensity of 40 μmol·m^−2^·s^−1^, Beijing Electronic Resource, Inc., China) was applied on the basis of the red light treatment, cycled through 18 h of light and 6 h of darkness, and deionized water was sprayed. During the germination phase of the aforementioned treatment, deionized water was sprayed in 40 mL increments every 12 h. On the second and fourth days of the treatment, seedlings were gathered for subsequent biochemical examination. Figure 1 summarizes the treatment schematic diagram of the effects of red light combined with UV-A and UV-B on black wheat seedlings.

### 2.2. Determination of Seedling Length and Fresh Weight

Thirty black wheat seedlings were randomly selected from each treatment group. Precise measurements of seedling length were obtained using a vernier caliper, while their fresh weights were accurately determined using an analytical balance.

### 2.3. Determination of Chlorophyll Content

The approach outlined by Zhang et al. [23] was utilized to measure the chlorophyll content in black wheat seedlings. Black wheat seedlings were homogenized with calcium carbonate powder and ethanol. Subsequently, acetone was added to the homogenate, and the mixture was shaken. The sample underwent centrifugation, resulting in the collection of the supernatant. The absorbances were then gauged at 645 nm and 663 nm.

### 2.4. Determination of Phenolic Acid Content

The approach outlined by Chen et al. [21] was utilized. The samples were ground with methanol, centrifuged after ultrasonication, and the supernatant was taken. The extraction was repeated three times. The supernatant was combined, dried, and dissolved in methanol to a constant volume as a free phenolic acid extract. The remaining residue was added to n-hexane, centrifuged to remove the supernatant, added to a sulfuric acid water bath, and then added to ethyl acetate for extraction. The extraction was repeated three times. After the extraction phase was combined, rotary evaporation was performed, and the residue was diluted with methanol as a bound phenolic acid extract. The mixture of phenolic acid extract and sodium dodecyl sulfate in a ferric chloride–potassium ferricyanide solution was diluted with methanol. The resulting mixture was incubated in the dark, then a hydrochloric acid solution was added and further incubated in the dark. Subsequently, the absorbance was measured at 760 nm, and the content of phenolic acid was determined according to the standard curve of gallic acid.

### 2.5. Determination of Total Phenolic, Total Flavonoid, and Anthocyanin Contents

The total phenolic content was determined following the protocols outlined by Mencin et al. [24]. The black wheat seedlings were pulverized using methanol followed by centrifugation to isolate the supernatant fraction. Subsequently, it was blended with Folin-phenol reagent and Na_2_CO_3_ and kept in darkness during incubation. The determination was performed at 765 nm and calculated according to the standard curve of gallic acid. The total flavonoid content was quantified following the methodology described by Yin et al. [25]. The black wheat seedlings were ground with ethanol, then subjected to ultrasonication followed by centrifugation to isolate the supernatant fraction. Then, diluted and quantitative absorbance measurements were taken at 260 nm. The anthocyanin content was quantified in accordance with the methodology proposed by Lao et al. [26]. Black wheat seedlings were ground in acidified ethanol followed by centrifugation and collection of the supernatant. The absorbance measurements were taken at 535 nm.

### 2.6. Determination of Malondialdehyde, Superoxide Anion, and Hydrogen Peroxide

The measurement of the malondialdehyde (MDA) content was conducted according to the method described by Zhuang et al. [27]. The levels of superoxide anion ($O_{2}^{-.}$) and hydrogen peroxide (H_2_O_2_) were quantified following the procedures detailed by Zhao et al. [28].

### 2.7. Determination of Antioxidant Enzyme Activity

The black wheat samples were ground in a phosphate-buffer solution (pH 7.0, 50 mM), followed by centrifugation to separate the supernatant fraction. The superoxide dismutase (SOD) and ascorbate peroxidase (APX) activities were quantified using the protocol established by Bin et al. [29]. A unit of SOD and APX activity was defined as a change of 0.01 per minute at OD_560nm_ and OD_290nm_, respectively. For the catalase (CAT) and peroxidase (POD) activities, the method of Yin et al. [30] was used. The units of the CAT and POD activities are defined as the change per minute at OD_240nm_ and OD_470nm_, respectively, with a value of 0.01.

### 2.8. Determination of Phenolic Acid Synthase Activity

The evaluation of PAL, C4H, and 4CL activities followed the protocol established by Yin et al. [30]. The black wheat seedlings were homogenized using Tris-HCl buffer (pH 8.9, 0.1 M) followed by centrifugation to separate the supernatant fraction. The activities of PAL, C4H, and 4CL are defined as one unit when the changes in OD_290nm_, OD_340nm_, and OD_333nm_ per minute are 0.01.

### 2.9. RNA Extraction and Quantitative Real-Time PCR Analysis

Total RNA was extracted from the black wheat seedlings employing the E.A.N.A.TM Plant RNA kit (R6827-01, OMEGA, Norcross, GA, USA). Subsequently, this RNA was reverse transcribed into cDNA employing the PrimeScript™ RT Master Mix Kit (RR036A, Takara, Japan). For quantitative analysis, triplicate reactions were performed on each cDNA sample, utilizing SYBR® Premix Ex Taq™ (RR420A, Takara, Japan) on a Light Cycler 480 II detection system (Roche, Basel, Switzerland). The oligonucleotide primers utilized for qRT-PCR are detailed in Supplementary Materials Table S1. The relative gene expression levels were determined employing the 2^−ΔΔCt^ method as described by Livak et al. [31].

### 2.10. Statistical Analysis

The results of the experiments are expressed as the means ± standard deviations based on triplicate measurements. A one-way ANOVA with Tukey’s multiple comparison tests was performed to evaluate the statistical significance at a predetermined level of 0.05 (*p* < 0.05).

## 3. Results

### 3.1. Effects of Red Light Combined with UV-A and UV-B Treatments on Physiological and Oxidative Damage Indexes of Black Wheat Seedlings

**Figure 2:**
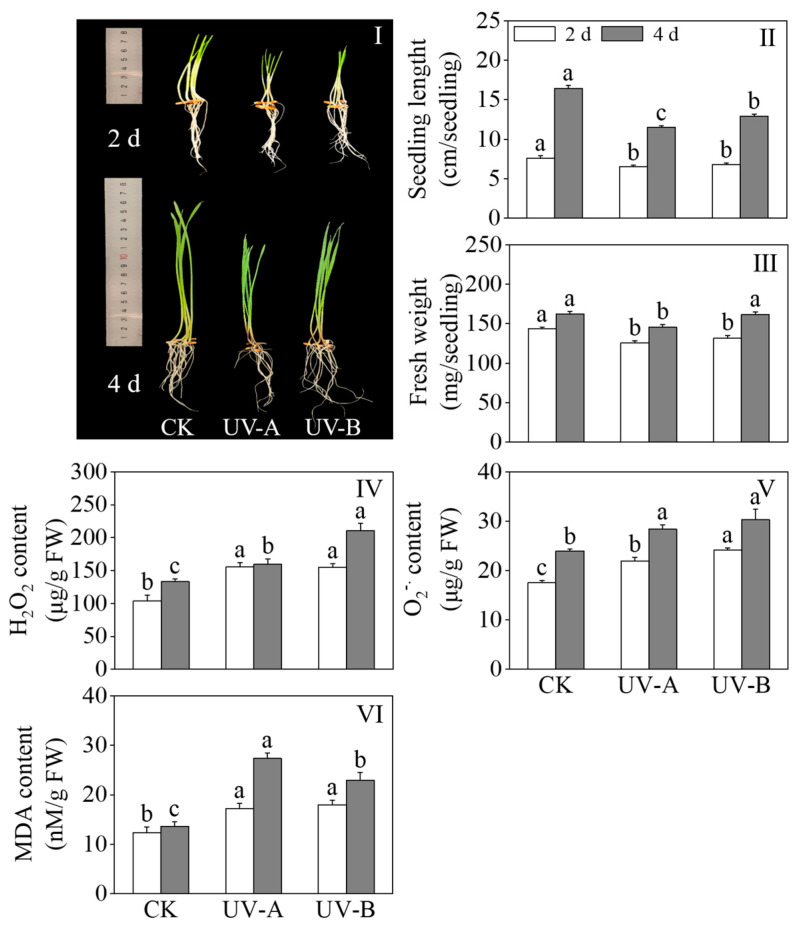
Effects of red light combined with the UV-A and UV-B treatments on the physiological and oxidative damage indexes of black wheat seedlings. The lowercase letters represent significant differences between different treatments at the same time.

Figure 2I–III show the effects of the red light combined with UV-A and UV-B treatments on the growth performance, seedling length, and fresh weight of black wheat seedlings. Compared with red light treatment alone, the UV-A and UV-B treatments retarded growth on black wheat seedlings. The length of black wheat seedlings treated with UV-A and UV-B dropped considerably (*p* < 0.05) to 70.12% and 78.66% of that treated with red light alone, respectively. The fresh weight of the UV-A treatment was lower than that of the other treatments after 4 days of light. The findings reveal that both the UV-A and UV-B treatments exhibited significant inhibitory impacts on the growth of black wheat seedlings, with UV-A demonstrating a stronger inhibitory effect. Figure 2IV–VI show the effects of the red light combined with UV-A and UV-B treatments on the H_2_O_2_ content, $O_{2}^{-}$ content, and MDA content of black wheat seedlings. The UV-A and UV-B treatments significantly enhanced the contents of H_2_O_2_, $O_{2}^{-}$, and MDA in black wheat seedlings compared with red light treatment alone (*p* < 0.05). Following four days of treatment, the H_2_O_2_ content in black wheat seedlings subjected to the UV-B treatment exhibited a notable elevation compared to those treated with UV-A (*p* < 0.05). After two days of light treatment, the content of $O_{2}^{-}$ in black wheat seedlings exposed to UV-B was notably elevated compared to those under UV-A, demonstrating a significant disparity (*p* < 0.05). In contrast, the MDA content remained relatively unchanged between the two treatments, lacking a notable disparity (*p* > 0.05). With the extension of the illumination time, there was no notable disparity in the $O_{2}^{-.}$ contents between the UV-A and UV-B treatments on the 4th day (*p* > 0.05). The content of MDA exhibited a marked elevation in comparison to UV-B (*p* < 0.05). This indicates that the UV-A and UV-B treatments resulted in varying degrees of oxidative damage to black wheat seedlings.

### 3.2. Impact of Red Light Combined with UV-A and UV-B Treatments on the Phenolic Acids, Total Phenols, Anthocyanin, and Total Flavonoid Contents of Black Wheat Seedlings

**Figure 3:**
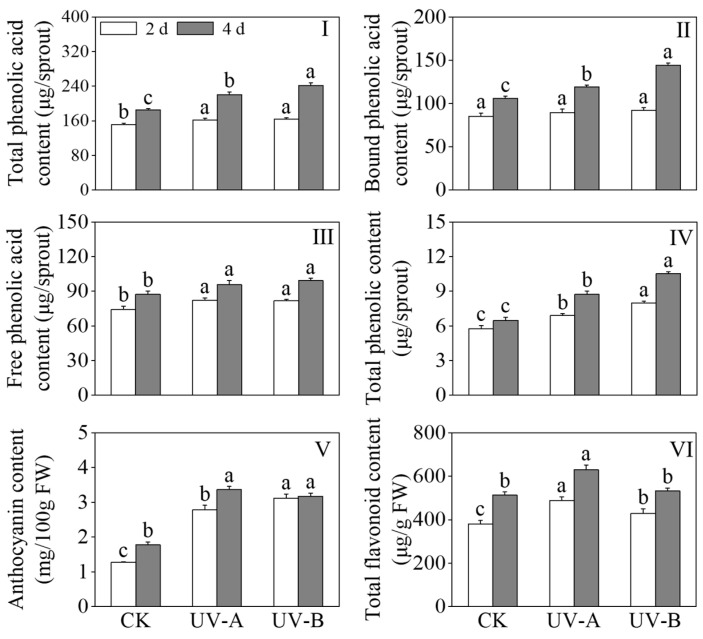
Effects of red light combined with the UV-A and UV-B treatments on the contents of phenols in black wheat seedlings. The lowercase letters represent significant differences between different treatments at the same time.

Figure 3 shows the effects of red light combined with UV-A and UV-B treatments on the contents of total phenolic acids, free phenolic acids, bound phenolic acids, total phenols, anthocyanins, and total flavonoids in black wheat seedlings. The results presented in Figure 2I–III depict the impact of red light combined with UV-A and UV-B treatments on the levels of phenolic acids in black wheat seedlings. Compared with the red light treatment alone, the total phenolic acid content of the UV-A and UV-B treatments increased by 19% and 30.4%, respectively, after 4 days of treatment. Following two days of UV-A and UV-B treatments, a substantial augmentation in the content of free phenolic acids was observed (*p* < 0.05), while the content of bound phenolic acids remained largely unchanged (*p* > 0.05). On the fourth day, the levels of both free and bound phenolic acids were found to be 1.14 and 1.36 times higher, respectively, in response to the UV-B treatment compared to the red light treatment. While exposure to UV-A treatment contributed to the enhancement of bound phenolic acid levels, its impact proved to be comparatively inferior to that induced by UV-B (*p* < 0.05). The contents of total phenols and anthocyanins in black wheat seedlings under the UV-A and UV-B treatments were notably elevated compared with those treated with red light, and the total phenolic content was significantly elevated under the UV-B treatment in contrast to the UV-A treatment (*p* < 0.05). After a four-day UV-A treatment, the total flavonoid content exhibited a notable elevation in contrast to the other treatment groups (*p* < 0.05). The above outcomes showed that although the UV-B treatment accumulated more phenolic substances, the promotion effect on total flavonoids was less than that of UV-A (Figure 2IV,VI).

### 3.3. Impact of Red Light Combined with UV-A and UV-B Treatments on Chlorophyll Content and Related Genes’ Expression Levels of Photosynthesis of Black Wheat Seedlings

**Figure 4:**
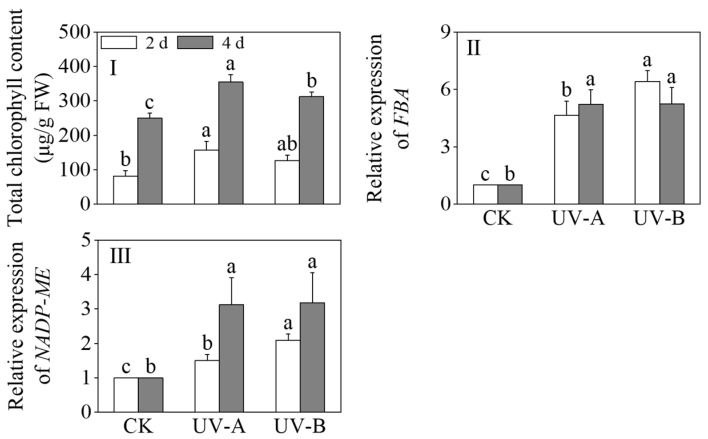
Effects of the red light treatment combined with UV-A and UV-B treatments on the chlorophyll contents and relative genes’ expression levels of photosynthesis in black wheat seedlings. The lowercase letters represent significant differences between different treatments at the same time.

Figure 4 shows the effects of red light combined with UV-A and UV-B treatments on the total chlorophyll content, *FBA*, and *NADP-ME* expression levels of black wheat seedlings. The chlorophyll contents exhibited a significant increase under the UV-A and UV-B treatments in contrast to the sole application of red light (*p* < 0.05). After 4 days, the chlorophyll contents under the UV-A and UV-B treatments were 1.57 times and 1.33 times that of red light alone, respectively. The chlorophyll levels following exposure to UV-A treatment exhibited a marked increase compared to those subjected to UV-B treatment (*p* < 0.05). UV-A and UV-B notably increased the relative expression levels of *fructose-1,6-bisphosphate aldolase* (*FBA*) and *NADP-malic enzyme* (*NADP-ME*) (*p* < 0.05). The relative expression level of *FBA* under the UV-A treatment gradually increased, and the relative expression level reached its highest at 4 days. During the UV-B treatment, the relative expression level of *FBA* peaked on the second day. After 2 days of light treatment, the relative expression level of *NADP-ME* peaked under the UV-B treatment, which was 2.09 times that of the red light treatment alone. At 4 days of light treatment, the relative expression levels of *NADP-ME* between the UV-A and UV-B treatments did not show a notable disparity (*p* > 0.05).

### 3.4. Impact of Red Light Combined with UV-A and UV-B Treatments on the Antioxidant Enzyme Activity and Relative Genes’ Expression Levels in Black Wheat Seedlings

**Figure 5:**
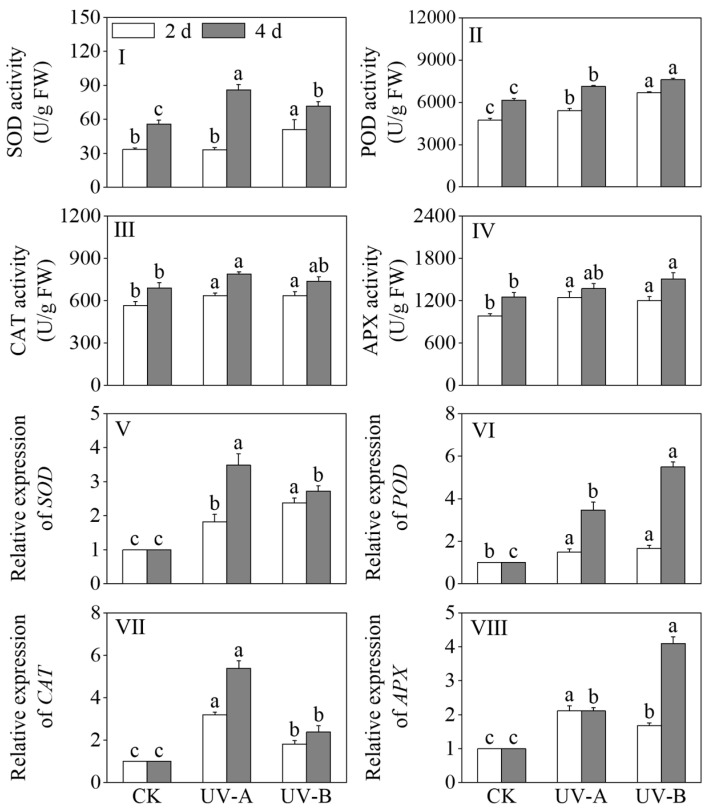
Effects of the red light treatment combined with UV-A and UV-B treatments on the antioxidant enzyme activity and relative gene expression levels in black wheat seedings. The lowercase letters represent significant differences between different treatments at the same time.

The effects of the red light treatment combined with UV-A and UV-B treatments on the activities of SOD, POD, CAT, and APX in black wheat seedlings are shown in Figure 5I–IV. After a four-day light treatment period, both the UV-A and UV-B treatments exhibited a notable augmentation in SOD and POD activities in comparison to the red light treatment (*p* < 0.05). The SOD activity increased 1.55-fold under the UV-A and 1.29-fold under the UV-B treatments compared to red light alone, while the POD activity rose by 15.83% and 23.79%, respectively. After 2 days of light treatment, under the UV-A and UV-B treatments, the CAT and APX activities were significantly elevated compared to those observed under the red light treatment (*p* < 0.05). In contrast to the red light treatment, the CAT activity increased by 12.64% and 12.95%, respectively. The APX activity was elevated 1.26-fold under the UV-A and 1.22-fold under the UV-B treatments compared with red light alone. This indicates that the red light treatment combined with UV-A and UV-B treatments activated the antioxidant system of the black wheat seedlings.

Figure 5V–VIII show the effects of the red light combined with UV-A and UV-B treatments on the expression levels of *SOD*, *POD*, *CAT*, and *APX* in black wheat seedlings. The UV-A and UV-B treatments notably increased the relative expression levels of *SOD*, *POD*, *CAT*, and *APX* in contrast to the red light treatment. (*p* < 0.05). The relative expression level of *SOD* was notably elevated under the UV-B treatment compared to UV-A (*p* < 0.05), while the relative expression levels of *CAT* and *APX* were notably higher under the UV-A treatment in contrast to UV-B (*p* < 0.05). The relative expression levels of *SOD* and *CAT* were significantly elevated under the UV-A treatment compared to the other treatments (*p* < 0.05), while the relative expression levels of *POD* and *APX* under the UV-B treatment were higher and 5.49 and 4.09 times that of the red light treatment, respectively. The results indicate that, as the illumination time increased, the UV-A treatment markedly upregulated the expression levels of *SOD* and *CAT* (*p* < 0.05), while the UV-B treatment exhibited a more potent inductive effect on the expressions of *POD* and *APX*.

### 3.5. Impact of the Red Light Combined with UV-A and UV-B Treatments on the Activities of Enzymes Related to Phenolic Acid Synthesis and the Relative Gene Expression Levels in Black Wheat Seedlings

**Figure 6:**
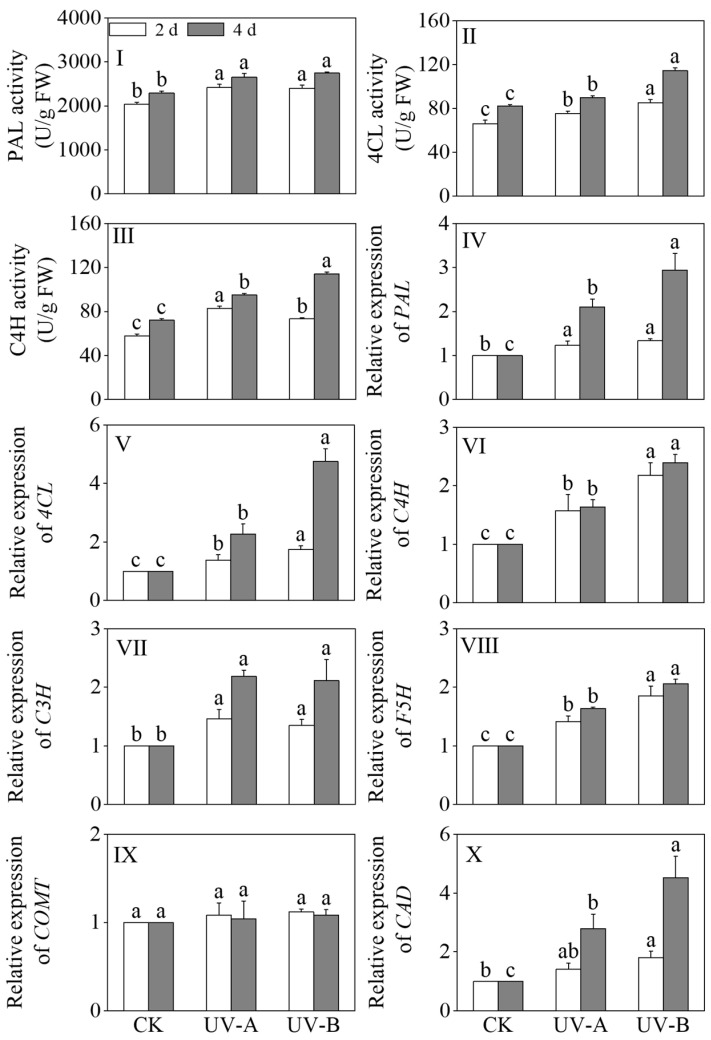
Effects of the red light combined with UV-A and UV-B treatments on the enzyme activities and relative genes’ expression levels of phenolic acid synthesis in black wheat seedlings. The lowercase letters represent significant differences between different treatments at the same time.

The effects of the red light combined with UV-A and UV-B treatments on the activities of PAL, 4CL, and C4H in black wheat seedlings were described in Figure 6I–III. Compared with the red light treatment, the PAL, 4CL, and C4H enzyme activities exhibited significant upregulation after 4 days of the UV-A and UV-B exposures (*p* < 0.05). Specifically, the UV-A treatment led to 1.16-, 1.09-, and 1.32-fold increases in the PAL, 4CL, and C4H activities, respectively, while the UV-B treatment resulted in 1.20-, 1.39-, and 1.58-fold enhancements. The 4CL and C4H activities were notably elevated under the UV-B treatment in contrast to the UV-A treatment (*p* < 0.05). 

The relative expression levels of *PAL*, *4CL*, *C3H*, *C4H*, ferulate 5-hydroxylase (*F5H*), *COMT*, and cinnamyl alcohol dehydrogenase (*CAD*) in the phenolic acid synthesis pathway are shown in Figure 6V–X. Upon prolonged treatment, the relative expression levels of key enzymes in the phenolic acid synthesis pathway, including *PAL*, *4CL*, *C3H*, *C4H*, *F5H*, and *CAD*, exhibited significant upregulation under the UV-A and UV-B treatments (*p* < 0.05). In black wheat seedlings exposed to the UV-A treatment for 4 days, the relative expression levels of *PAL*, *4CL*, *C3H*, *C4H*, *F5H*, and *CAD* were upregulated by 2.10-, 2.30-, 2.18-, 1.64-, 1.64-, and 2.79-fold, respectively. In black wheat seedlings subjected to UV-B treatment for 4 days, the relative expression levels of *PAL*, *4CL*, *C3H*, *C4H*, *F5H*, and *CAD* underwent marked upregulation, specifically by 2.94, 4.75, 2.11, 2.39, 2.06, and 4.53 times, respectively. This indicated that the UV-B treatment had a more notable upregulation effect on the relative expression levels of *PAL*, *4CL*, *C4H*, *F5H,* and *CAD*. In addition, the relative expression level of *caffeic acid O-methyl transferase* (*COMT*) under the light treatment did not change significantly compared with the red light treatment (*p* > 0.05).

## 4. Discussion

When plants are subjected to UV radiation for an extended period of time, they show a variety of morphological and physiological reactions, which may lead to cell damage. This damage may include DNA damage, protein damage, and cell membrane structure damage, which might hinder plant growth, development, and viability [22,32]. In this study, the impacts of the red light combined with UV-A and UV-B treatments on the accumulation of phenolic acids in black wheat seedlings was investigated, and the impacts of these treatments on the physiology and biochemistry of black wheat seedlings was also explored. The outcomes reveal that the UV-A and UV-B treatments caused a decrease in the seedling length of the black wheat seedlings and an increase in the contents of $O_{2}^{-.}$, H_2_O_2_, and MDA, the main markers of plant membrane lipid peroxidation. This indicates that the UV-A and UV-B treatments induced a certain degree of oxidative damage in the black wheat seedlings, and the phenolic acid content in the black wheat seedlings significantly increased under the UV-A and UV-B treatments.

The enrichment of phenolic acids is linked to the regulation of the activities and gene expression levels of PAL, 4CL, C4H, C3H, COMT, F5H, and CAD via the biosynthetic route. Prohydrojasmon treatment led to a substantial upregulation in the relative expression levels of *PAL* and *F3H* in lettuce, thereby significantly augmenting the levels of phenolic acids [13]. Under NaCl treatment, the activities and gene expression levels of PAL, 4CL, and C4H in barley were increased, which promoted the biosynthesis of more phenolic acids [12]. The findings of this study unmistakably indicate that both UV-A and UV-B treatments led to oxidative damage in the interior of black wheat seedlings. This damage may act as a signal to trigger the activation of the phenolic acid synthesis pathway [32]. Specifically, both UV treatments markedly elevated the activities of PAL, 4CL, and C4H, three key phenolic acid synthases. However, through comparative analysis, it was discovered that the effect of the UV-B treatment on the augmentation of the activities of these enzymes was notably greater than that of the UV-A treatment. It is worth noting that the UV-B treatment not only showed a stronger induction at the enzyme activity level but also showed a similar trend at the gene expression level. The relative expression levels of the enzymes *PAL*, *4CL*, *C4H*, *F5H*, and *CAD* were observed to be substantially elevated following the UV-B treatment compared to those under the UV-A treatment. This indicates that the UV-B treatment was stronger than the UV-A treatment in inducing the activation of the phenolic acid synthesis pathway. This effect is not only reflected in the enzyme activity but also further verified at the level of gene transcription. The above findings reveal that the induction impact of the UV-B treatment on the expression of the phenolic acid synthase gene in black wheat seedlings was stronger than that of the UV-A treatment. The relative expression levels of *COMT* in black wheat did not change significantly under the UV-A and UV-B treatments. This may be due to the short lengths of time for the red light combined with UV-A and UV-B treatments, which had little impact on the expression of *COMT* [19].

As a part of the solar spectrum, UV-A and UV-B radiations have a marked impact on plant photosynthesis. In this study, compared with the red light treatment alone, the red light combined with UV-A and UV-B treatments significantly elevated the levels of photosynthetic pigments in black wheat seedlings. An increase in photosynthetic pigments usually means an increase in the light energy utilization rate of plants, which is very important for improving the photosynthetic efficiency of plants. FBA and NADP-ME are two important enzymes for photosynthesis. Under the action of UV-A and UV-B, the relative expression levels of *FBA* and *NADP-ME* were significantly elevated compared to those observed under the red light treatment alone. *FBA* and *NADP-ME* occupy pivotal positions in the light and dark reactions of photosynthesis. The increase in their gene expression may directly promote photosynthesis, thus improving the photosynthetic efficiency of black wheat seedlings [33,34]. It bears mentioning that the UV-A and UV-B treatments not only enhance the photosynthesis of black wheat seedlings but may also improve their antioxidant properties. This may be because the energy and substances produced by photosynthesis can provide antioxidants for plants and help them resist oxidative damage caused by UV-A and UV-B [35].

UV-A and UV-B treatments can lead to an elevation in the formation of ROS in plant cells, resulting in oxidative damage and injury to cell structure and function [36]. In order to cope with this challenge, plants have developed an efficient antioxidant enzyme system as one of the most important means of alleviating oxidative damage [37]. Studies have shown that plants can increase their seedlings endurance to UV duress by upregulating the activity of antioxidant enzymes [38]. In this study, it was observed that the POD, SOD, CAT, and APX activities in black wheat seedlings were significantly enhanced under the red light combined with UV-A and UV-B treatments, which were significantly different from that under the red light treatment. Simultaneously, the gene expression levels of these antioxidant enzymes were also significantly increased under compound light treatment. This result clearly shows that black wheat seedlings can effectively mobilize their antioxidant enzyme system to cope with and alleviate the environmental pressures caused by UV-A and UV-B stress.

**Figure 7:**
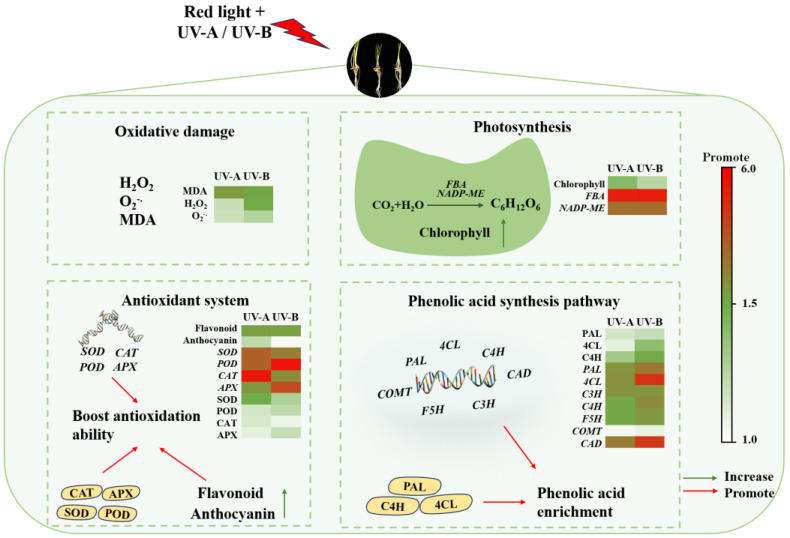
Hypothesis of the mechanisms of the red light combined with UV-A and UV-B treatments on black wheat seedling growth and the regulation of phenolic acid enrichment.

Phenolic substances, comprising an indispensable class of plant secondary metabolites, serve as vital components of plants’ nonenzymatic antioxidant system [36]. In this study, it was found that when black wheat seedlings were treated with red light combined with UV-A and UV-B, the contents of total flavonoids and anthocyanins in their bodies increased significantly. The findings indicate that UV-A and UV-B irradiation promoted the secondary metabolic activity of black wheat seedlings and induced the synthesis of antioxidant substances. Phenolic compounds, specifically total flavonoids and anthocyanins, exert a crucial function in eliminating free radicals, thereby mitigating oxidative damage to cells. Under UV-A and UV-B stress, black wheat seedlings enhanced their own defense mechanism by increasing the synthesis of these antioxidants, thus mitigating the harm due to UV radiation. The results are consistent with the conclusions of Chen et al. [21], who observed that the phenolic compounds in wheat seedlings were elevated after the UV-B treatment alone. It was also found that the UV-A treatment could markedly elevate the content of phenols in strawberries [16] and broccoli [17]. In addition, in this research, the anthocyanin content under the UV-B treatment was notably greater than that achieved under the UV-A treatment, while the total flavonoid content was significantly elevated under the UV-A treatment in contrast to the UV-B treatment, which may be related to the differences between UV-B and UV-A in wavelength, energy distribution, and mechanism of action on plant cells [38]. Based on the above discussion, Figure 7 summarizes the mechanism of the impact of irradiation from a light source on the enrichment and physiological metabolism of phenolic acids in black wheat seedlings.

## 5. Conclusions

This study investigated the regulatory effects of red light combined with UV-A and UV-B treatments on the synthesis of phenolic acids in black wheat seedlings. The results show that red light combined with UV-A and UV-B significantly enhanced the activities of PAL, C4H, and 4CL and differentially upregulated the relative gene expression levels of phenolic acid synthesis, thus promoting phenolic acid synthesis. UV-B exhibited a more significant enrichment effect. Furthermore, the UV-A and UV-B treatments increased the chlorophyll content, upregulated the relative gene expression levels of photosynthesis, and stimulated photosynthesis in black wheat seedlings. Additionally, UV-A and UV-B treatments suppressed the growth and development of black wheat seedlings, activated the antioxidant system, and improved the antioxidant performance of black wheat seedlings. The red light combined with UV-A and UV-B treatments play a positive role in promoting phenolic acid accumulation in black wheat seedlings, providing theoretical support and practical guidance for the production of black wheat products rich in phenolic acids.

## Data Availability

The original contributions presented in the study are included in the article/Supplementary Material, further inquiries can be directed to the corresponding author.

## Supplementary Materials

The following supporting information can be downloaded at: https://www.mdpi.com/article/10.3390/foods13162499/s1, Table S1: Sequence-specific primers used in the present study.
