# Supplementary material for: The Impact of Lighting Treatments on the Biosynthesis of Phenolic Acids in Black Wheat Seedlings
Source: Foods. 2024 Aug 9;13(16):2499. doi: 10.3390/foods13162499 (PMC11353367; doi:10.3390/foods13162499)
Supplement: Supplementary file 1 [file foods-13-02499-s001.zip › foods-3117908-supplementary.pdf]

Supplement Table S1. Sequence-specific primers used in the present study.

	Gene name	Forward Primer (5' to 3')	Reverse Primer (5' to 3')
1	Actin	AGCGGTCGAACAACTGGTA	AAACGAAGGATAGCATGAGGAAGC
2	*PAL*	CACCACCCTGGACAGATTG	TGAGGCGAAGTGCGGAG
3	*4CL*	ACATTACACAAGCAGGAAGAACC	CACTCAGCCAGCCAGCAG
4	*C3H*	ATTGACGAAGAAGGGCAG	GGACACAGCCATCTCAAGT
5	*C4H*	CAGCCTCCACATCCTCAAG	CTTAGGACGAGCGAACAATC
6	*F5H*	AGCTCCCCTCTCTCAAGTGC	GACACAGTCCTCGGCGTTCT
7	*COMT*	ACGCTGCTCAAGAACTGCT	CGGGTTCACAGGCAGGAT
8	*CAD*	CTGCTCAAGGTGAACGGGAA	CATCATCTCCTGCGTCTCCTTC
9	*POD*	GACCAGGTGCTCTTCAACAACGAC	TAGCCGTAGGTCAATCACGAGTTC
10	*SOD*	GAGTAGAAATCCGCTCCCGAC	GAGGCAGGAACCTGGAAGAGA
11	*APX*	CAGCAATGTGTGAGGCTTGGC	CAATGATGACACCGTCGGTGG
12	*FBA*	ATCGACCATGTCGGCCTA	ATATATCAGGACTCAAAACCAAAC
13	*NADP-ME*	GAAGCATACAAATGGACCAAGG	CAAGAACAGCGACAGACAACAA
14	*CAT*	CCTTCCTCTTCGACGACATC	CCGTCGTAGTGGTTGTTGTG
